# Tangential biopsy angle and needle depth for adequacy and safety outcomes in ultrasound-guided native kidney biopsy—a single-center experience in a high-risk population

**DOI:** 10.1007/s40620-025-02362-x

**Published:** 2025-07-29

**Authors:** Ittikorn Spanuchart, Thanaporn Supachokchaiwattana, Kanin Thammavaranucupt, Kaewpitcha Pichitpichatkul, Suchin Worawichawong, Chinnarat Bua-ngam

**Affiliations:** 1 Division of Nephrology, Department of Internal Medicine, University of Michigan, Ann Arbor, USA; 2 Division of Nephrology, Department of Internal Medicine, Faculty of Medicine Ramathibodi Hospital, Mahidol University, Bangkok, Thailand; 3 Division of Body Interventional Radiology, Department of Diagnostic and Therapeutic Radiology, Faculty of Medicine Ramathibodi Hospital, Mahidol University, Bangkok, Thailand; 4 Division of Diagnostic Radiology, Department of Diagnostic and Therapeutic Radiology, Faculty of Medicine Ramathibodi Hospital, Mahidol University, Bangkok, Thailand; 5 Chakri Naruebodindra Medical Institute, Faculty of Medicine Ramathibodi Hospital, Mahidol University, Samut Prakan, Thailand; 6 Department of Pathology, Faculty of Medicine Ramathibodi Hospital, Mahidol University, Bangkok, Thailand

**Keywords:** Ultrasound-guided, Biopsy, Native kidney, Tangential angle

## Abstract

**Background:**

Kidney biopsy is crucial for diagnosing kidney diseases but involves risks, notably bleeding, which must be balanced with diagnostic precision. This study examines the effect of the biopsy needle’s cortical tangential angle and depth on specimen adequacy and safety outcomes.

**Methods:**

This single-center, retrospective study reviewed electronic medical records from kidney biopsies performed between January 1, 2016 and December 31, 2020. Included were patients undergoing real-time ultrasound-guided percutaneous kidney biopsies. Exclusion criteria were pediatric patients, renal mass or transplant biopsies, and cases with incomplete records. Primary variables included biopsy needle cortical tangential angle and depth. Outcomes were tissue adequacy and safety, with complications assessed within 24 h.

**Results:**

Out of 443 biopsies performed, 124 met the inclusion criteria. Our patient population had a mean BMI of 27.17 kg/m^2^, which met the criteria for obesity based on BMI standards for Asians, and they also had relatively small kidneys (< 9 cm) with parenchymal thinning. Biopsies at angles of 30°–60° yielded more glomeruli (12 vs. 5, *p* < 0.001) and had a higher pathologist-reported adequacy (82.67% vs. 59.18%, *p* = 0.004). Needle depth did not significantly impact adequacy. Major complications occurred in 12.90% of cases, with blood transfusions required in 8.06% and embolizations in 3.23%. All technical factors lost statistical significance after adjusting for confounders, except for increased echogenicity, which remained significant.

**Conclusions:**

The optimal needle angle for kidney biopsies is 30°–60° for the highest diagnostic yield compared to angles < 30° or > 60°. Our study did not reveal statistically significant differences in major complications between these angle ranges. This greater understanding of the relationship between biopsy angle, needle trajectory depth, and diagnostic and safety outcomes offers valuable insights for optimizing kidney biopsy procedures.

**Graphical Abstract:**

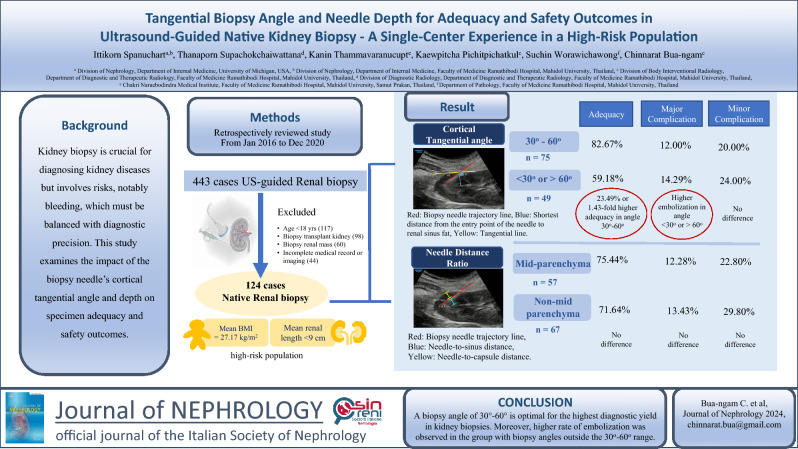

## Introduction

Kidney biopsy is a vital diagnostic tool for assessing various kidney diseases, providing crucial insights for clinical decision-making [1]. Acquiring a sufficient number of glomeruli is essential for establishing a definitive diagnosis. Despite the importance of obtaining ample tissue to enhance diagnostic precision, kidney biopsy involves potential risks, especially bleeding, necessitating a delicate balance between optimizing diagnostic yield and minimizing these risks.

Well-established risk factors for kidney biopsy-related bleeding include uncontrolled hypertension, anemia, coagulopathy, and elevated serum creatinine [2]. Conditions like obesity and chronic kidney disease, characterized by small kidney size or thin kidney parenchyma, introduce additional complexities, making procedural technical aspects more challenging.

Recent attention has focused on studying technical factors associated with kidney biopsy [3–6]. The real-time ultrasound-guided approach, particularly the cortical tangential method, emerges as a potential strategy to optimize procedural success and reduce bleeding complications. However, optimal biopsy angles and needle depth, balancing between the highest diagnostic yield and lowest complications, remain controversial issues [7].

Our study sought to evaluate the influence of the biopsy needle cortical tangential angle and biopsy needle depth on specimen adequacy and safety outcomes.

## Methods

### Study design

This is a single-center, retrospective, cross-sectional investigation approved by the Human Research Ethics Committee of the Faculty of Medicine Ramathibodi Hospital, Mahidol University (MURA2020/1652) prior to the study's commencement. The electronic medical records of patients who underwent kidney biopsies performed by interventional radiologists at the Department of Radiology, Ramathibodi Hospital, were reviewed from January 1, 2016 to December 31, 2020.

### Study population

The study included all patients who underwent real-time ultrasound-guided percutaneous kidney biopsies by interventional radiologists at the Department of Radiology, Ramathibodi Hospital, from January 1, 2016 to December 31, 2020. Exclusion criteria were pediatric patients (< 18 years of age), renal mass biopsies, transplant kidney biopsies, and cases with incomplete ultrasound images or unavailable medical records.

### Biopsy procedure

All biopsies were performed or supervised by experienced interventional radiologists using real-time ultrasound guidance (iU22, Philips, USA and RS85, Samsung Medison, Seoul, Korea) and the freehand technique in a prone position for native kidney biopsy. Tissue samples were obtained with a 16-gauge automated spring-loaded biopsy device featuring a 2 cm core length [Achieve™ (Merit medical system, Utah, USA) or Medone (MEDAX srl Unipersonale, Poggio Rusco, Italy)]. Ultrasound images captured the needle trajectory line, and immediate complications were assessed. Following the procedure, patients underwent a 24-h observation period before discharge.

### Study explanatory variables

The primary explanatory variables in this study were the biopsy needle cortical tangential angle and the biopsy needle depth. Additional explanatory variables included the biopsy plane, the needle direction, and the biopsy site of the kidney.

**Fig. 1:**
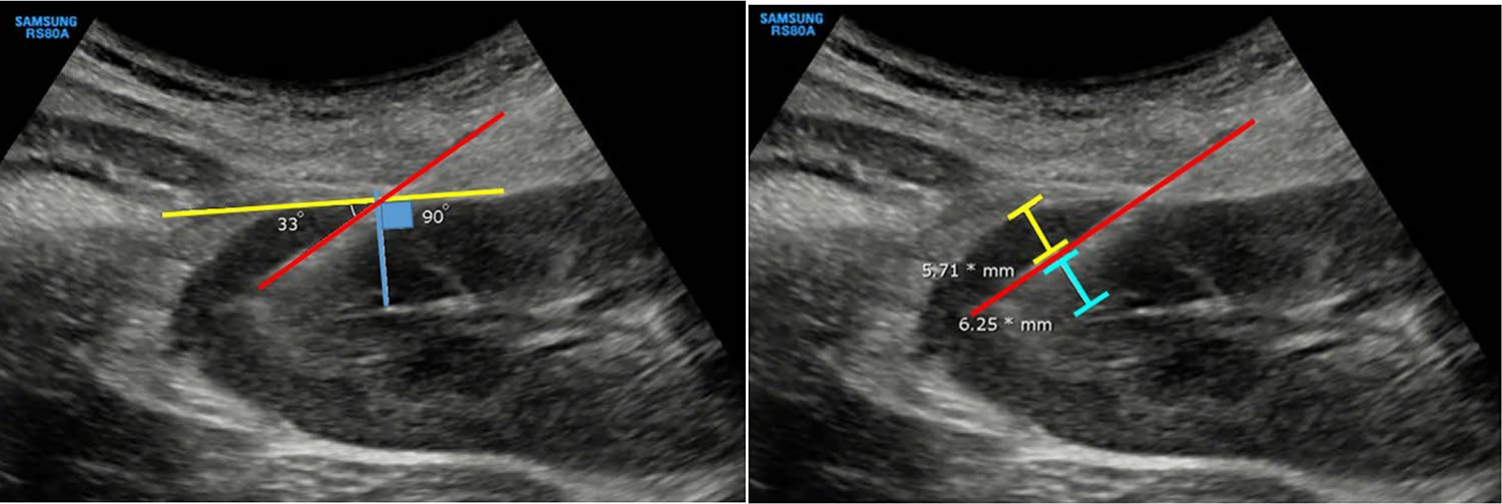
Cortical tangential angle (left)—Red: Biopsy needle trajectory line, Blue: Shortest distance from the entry point of the needle to renal sinus fat, Yellow: Tangential line. Needle Distance Ratio (right)—Red: Biopsy needle trajectory line, Blue: Needle-to-sinus distance, Yellow: Needle-to-capsule distance

The biopsy needle cortical tangential angle was defined as the angle between the needle trajectory and tangential lines. The tangential line was drawn perpendicular to the line extending from the point of needle entry into the renal parenchyma to renal sinus fat in the shortest distance (Fig. 1). For analytical purposes, biopsy angles were categorized into two groups: 30°–60° and < 30° or > 60°, with the former representing an angle usually aimed for in common practice.

The biopsy needle depth was determined using the needle distance ratio, calculated as the ratio of the needle-to-capsule distance to the total distance from capsule to sinus (needle-to-capsule distance + needle-to-sinus distance). The needle-to-capsule distance was measured as the furthest distance from the needle trajectory line to the renal capsule in the perpendicular dimension, while the needle-to-sinus distance represented the nearest distance between the needle trajectory line and the renal sinus (Fig. 1). Needle trajectory depth was categorized into four groups based on the needle distance ratio: outer parenchyma (0–0.33), middle parenchyma (0.34–0.66), inner parenchyma (0.67–0.99), and the sinus group (1.0), with the sinus group representing trajectories extending beyond the parenchyma into the renal sinus. For analytical purposes, the mid-parenchyma group (0.34–0.66) was compared to the non-mid- parenchyma group, which combined the outer, inner, and sinus groups.

The biopsy planes were categorized as the longitudinal plane, where the ultrasound probe was placed perpendicular to the kidney to capture the maximum distance from pole to pole, and the oblique plane, characterized by tilting the ultrasound probe away from the longitudinal plane.

The biopsy needle directions were divided into caudal and cranial directions. The caudal direction was determined when the biopsy needle tip pointed towards the legs, while the cranial direction was identified when the biopsy needle tip was directed towards the head.

The biopsy sites of the kidney were categorized as the lower pole, middle pole, and upper pole. For analytical purposes, the biopsy site was dichotomized into two groups: the lower pole and the combined upper/middle pole.

### Study outcomes

The primary outcome was tissue adequacy. Adequacy was the criterion used, as defined by the presence of at least 10 glomeruli in the specimens processed for light microscopy examination [8]. Suboptimal was defined for specimens containing at least 1 glomerulus but failing to meet the adequacy criteria. Inadequacy was attributed to specimens that did not satisfy the suboptimal criteria. Notably, all kidney pathology slides underwent a thorough review and diagnosis by a single renal pathologist, CW, within our institution.

The secondary outcome was the safety profile. Major complications were defined as any of the following events occurring within 24 h after the biopsy: hematoma size > 5 cm or requiring intervention (such as transfusion, endovascular or surgical intervention, or prolonged hospitalization), injury to the adjacent internal organ, acute renal obstruction, acute kidney injury, or death [9]. Minor complications were defined as any of the following events within 24 h after the biopsy: hematomas not requiring further intervention, gross hematuria without further intervention, and pain at the biopsy site.

### Data collection

The electronic medical records of enrolled patients underwent retrospective review. Collected information included patients' demographics, comorbidities, medications, laboratory results, kidney ultrasound parameters, kidney biopsy procedure-related data, and kidney pathological data. Measurements for kidney ultrasound and procedure-related data were conducted, and consensus was reached among the authors, TS and CB.

### Statistical analysis

Categorical data were presented as counts and percentages, while continuous data were reported as mean with standard deviation for parametric parameters and median with interquartile range (IQR) for non-parametric parameters. Differences were assessed using the Chi-square or Fisher exact test for categorical variables and the Student *t*-test or Mann–Whitney test for continuous variables.

Univariable logistic regression analysis was conducted to explore associations between each variable and the outcome variables. Potential confounders were included in a multivariable logistic regression model to adjust for their effects. The statistical analysis was performed using Stata, version 17.0 (StataCorp LP, College Station, TX, USA).

## Results

### Patient demographics, kidney ultrasound characteristics, and kidney biopsy procedure data

**Fig. 2:**
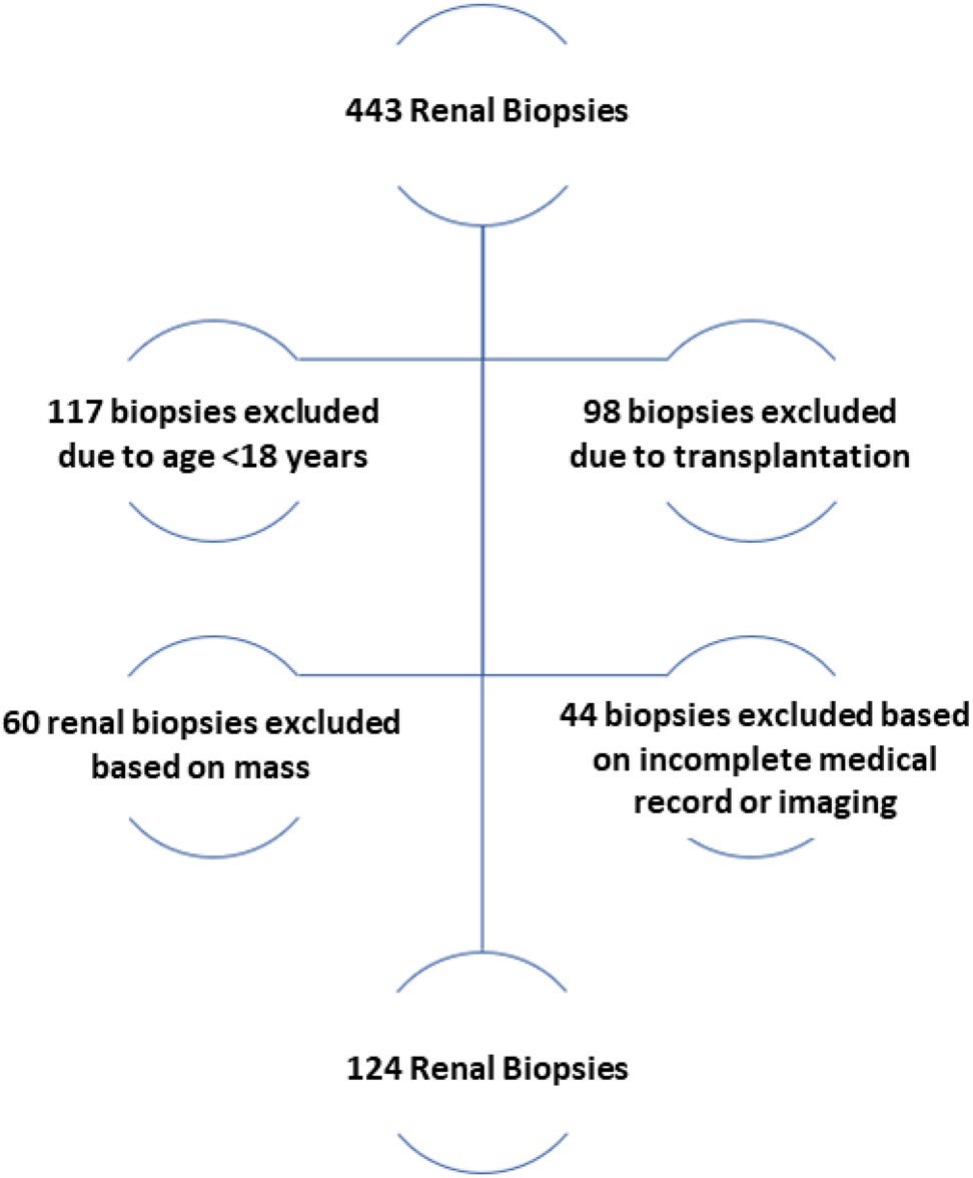
CONSORT diagram

Out of the 443 kidney biopsies performed by the interventional radiologists at our institution, 319 were excluded based on the criteria, and 124 were enrolled in the study (Fig. 2). The characteristics of the 124 patients, categorized by the biopsy needle tangential angle and the biopsy needle depth, are presented in Table 1.

**Table 1:** Patient’s baseline characteristics

	Biopsy needle tangential angle (*N*, %)			Biopsy needle depth (*N*, %)		
	30°–60^o^ (75, 60.48%)	< 30° or > 60^o^ (49, 39.52%)	*P*-value	Mid-parenchyma (57, 45.97%)	Non-mid-parenchyma (67, 54.03%)	*P*-value
**Patient characteristics**						
Age (years), mean ± SD	50.55 ± 18.29	52.98 ± 17.33	0.461	52.81 ± 18.00	50.40 ± 17.85	0.458
Gender, female, *N* (%)	34 (45.33)	19 (38.78)	0.471	26 (45.61)	27 (40.30)	0.551
Body mass index (kg/m^2^), mean ± SD	26.14 ± 6.42	28.74 ± 8.35	0.067	25.93 ± 7.33	28.22 ± 7.22	0.083
Hypertension, *N* (%)	54 (72.00)	38 (77.55)	0.490	41 (71.93)	51 (76.12)	0.595
Diabetes, *N* (%)	21 (28.00)	12 (24.49)	0.665	14 (24.56)	19 (28.36)	0.634
Heart disease, *N* (%)	10 (13.33)	4 (8.16)	0.374	6 (10.53)	8 (11.94)	0.804
Cirrhosis, *N* (%)	3 (4.00)	3 (6.12)	0.680	2 (3.51)	4 (5.97)	0.686
Vasculitis, *N* (%)	16 (21.33)	7 (14.29)	0.324	16 (28.07)	7 (10.45)	0.012*
Kidney replacement therapy, *N* (%)	13 (17.33)	8 (16.33)	0.884	7 (12.28)	14 (20.90)	0.202
Systolic blood pressure (mmHg), mean ± SD	136.8 ± 21.33	138.49 ± 16.75	0.641	139.84 ± 20.36	135.45 ± 18.83	0.215
Diastolic blood pressure (mmHg), mean ± SD	79.59 ± 12.92	79.12 ± 11.37	0.838	80.40 ± 12.30	78.55 ± 12.30	0.405
Use of antiplatelet/anticoagulant, *N* (%)	1 (1.33)	1 (2.04)	1.000	1 (1.75)	1 (1.49)	1.000
**Labs**						
Hemoglobin (mg/dL), mean ± SD	11.66 ± 2.37	11.4 ± 2.28	0.857	11.55 ± 2.25	11.81 ± 2.40	0.537
Platelets (× 1000 mm^3^), median (Q1, Q3)	235 (198, 289)	236 (202, 294)	0.749	244.68 ± 89.36	256.94 ± 83.85	0.433
International normalized ratio (INR), mean ± SD	0.97 ± 0.10	0.97 ± 0.09	0.961	0.96 ± 0.10	0.97 ± 0.10	0.410
Serum creatinine (mg/dL), median (Q1, Q3)	1.83 (1.18, 3.11)	2.08 (1.32, 3.96)	0.377	1.57 (0.9, 3.03)	2.3 (1.47, 4)	0.009*
eGFR (mL/min/1.73m^2^), median (Q1, Q3)	38.45 (19.5, 70.7)	33.8 (14.8, 53.4)	0.363	45.2 (22.7, 77.7)	28.6 (14.3, 51.1)	0.013*
**Ultrasound Characteristics**						
Kidney length (cm), mean ± SD	9.91 ± 1.61	9.79 ± 1.71	0.692	10.05 ± 1.51	9.70 ± 1.75	0.240
Kidney parenchymal thickness (mm), mean ± SD	13.86 ± 3.13	13.08 ± 3.54	0.199	14.04 ± 3.34	13.14 ± 3.25	0.135
Increased kidney echogenicity, *N* (%)	54 (72.00)	37 (75.51)	0.665	39 (68.42)	52 (77.61)	0.248
**Kidney Biopsy Procedure Data**						
Biopsy plane, *N* (%)						
Longitudinal plane	49 (65.33)	28 (57.14)	0.358	33 (57.89)	44 (65.67)	0.374
Oblique plane	26 (34.67)	21 (42.86)		24 (42.11)	23 (34.33)	
Biopsy site, *N* (%)						
Lower pole	67 (89.33)	46 (93.88)	0.524	48 (84.21)	65 (97.01)	0.012*
Upper or middle pole	8 (10.67)	3 (6.12)		9 (15.79)	2 (2.99)	
Biopsy needle direction, *N* (%)						
Caudal direction	51 (68.00)	41 (83.67)	0.051	39 (68.42)	53 (79.10)	0.175
Cranial direction	24 (32.00)	8 (16.33)		18 (31.58)	14 (20.90)	
Biopsy needle tangential angle, *N* (%)						
30°–60°				44 (77.19)	31 (46.27)	< 0.001*
< 30° or > 60°				13 (22.81)	36 (53.73)	
Biopsy needle distance ratio						
Mid-parenchyma (0.34–0.66)	44 (58.67)	13 (26.53)	< 0.001*			
Non-mid-parenchyma (< 0.34 or > 0.66)	31 (41.33)	36 (73.47)				

Baseline characteristics were comparable across all groups. All patients in our study were Asian. The mean age of the patients was 51.51 years, with 53 (42%) being female, and the mean BMI was 27.17 kg/m^2^. The most common comorbidities observed were hypertension (74.19%) and diabetes (26.61%). Notably, there were more patients with vasculitis in the group of biopsy needles in the middle zone, and creatinine levels were slightly higher in the inner/outer zone group. Twenty-one patients (16.94%) were receiving dialysis at the time of biopsies.

The mean kidney length was 9.86 cm, with a mean parenchymal thickness of 13.6 mm. Increased echogenicity was noted in 73.39% of cases, with 44.35% exhibiting a loss of corticomedullary differentiation. It is noteworthy that a significant portion of our patient population met the criteria for obesity based on BMI standards for Asians [10] and had relatively small kidneys with parenchymal thinning.

The majority of our patients underwent kidney biopsies in the longitudinal plane (62.10%), with the biopsy needle directed in the caudal direction (74.19%), primarily targeting the lower pole (91%). Most of the biopsies were performed at the angle of 30°–60° (75 biopsies, 60.48%) and in the middle zone with a needle distance ratio of 0.34–0.66 (57 biopsies, 45.97%). Notably, 32 (25.81%) biopsies passed across the renal sinus with a needle distance ratio of 1.

### Tissue adequacy and safety outcomes

The outcomes of tissue adequacy and safety are detailed in Table 2. Biopsies within the angle of 30°–60° yielded higher glomeruli (12 vs. 5, p < 0.001) and had a higher adequacy rate based on the number of glomeruli criteria (64% vs. 16%, *p* < 0.001) and from the pathologist's perspective (82.67% vs. 59.18%, *p* = 0.004). There was a trend towards higher adequacy in the group with needle biopsy in the middle zone compared to the inner/outer zone, but it was not statistically significant. Upon adjustment for potential confounders, only the biopsy angle of 30°–60° retained significance for the adequacy outcome with an odds ratio (OR) of 11.21 (95% CI of 3.74–33.62, *p* < 0.001), while the biopsy needle depth was not significant (Table 3). No statistically significant differences were observed between tissue adequacy and other technical factors, including the biopsy plane, the biopsy needle direction, and the biopsy site.

**Table 2:** Specimen adequacy and safety outcomes

Patient characteristics	Biopsy needle tangential angle (*N*, %)			Biopsy needle depth (*N*, %)		
	30°–60^o^ (75, 60.48%)	< 30° or > 60^o^ (49, 39.52%)	*P*-value	Mid-parenchyma (57, 45.97%)	Non-mid-parenchyma (67, 54.03%)	*P*-value
**Specimen Adequacy**						
Numbers of specimen cores, mean ± SD	2.07 ± 0.41	1.98 ± 0.38	0.240	2.07 ± 0.37	2.00 ± 0.43	0.335
Numbers of total glomeruli, median (Q1–Q3)	12 (6–17)	5 (3–9)	< 0.001*	10 (2–15)	8 (3–11)	0.089
Adequacy, glomeruli ≥ 10, *N* (%)	48 (64)	8 (16)	< 0.001*	30 (52.63)	26 (38.81)	0.123
Adequacy from pathologist perspective, *N* (%)	62 (82.67)	29 (59.18)	0.004*	43 (75.44)	48 (71.64)	0.634
**Complications**						
Major complications, *N* (%)	9 (12.00)	7 (14.29)	0.711	7 (12.28)	9 (13.43)	0.849
Blood transfusion	6 (8.00)	4 (8.16)	1.000	5 (8.77)	5 (7.46)	1.000
Embolization	0 (0)	4 (8.16)	0.023*	0 (0)	4 (5.97)	0.124
Nephrectomy	0 (0)	0 (0)		0 (0)	0 (0)	
Adjacent organ injury	1 (1.33)	0 (0)	1.000	0 (0)	1 (1.49)	1.000
Death	0 (0)	0 (0)		0 (0)	0 (0)	
Any complications, *N* (%)						
Hematoma, any size	31 (41.33)	18 (36.73)	0.609	19 (33.33)	30 (44.78)	0.194
Hematuria	4 (5.33)	8 (16.33)	0.061	4 (7.02)	8 (11.94)	0.355
Pain	11 (14.67)	10 (20.41)	0.405	9 (15.79)	12 (17.91)	0.754
AVF	17 (22.67)	8 (16.33)	0.494	13 (22.81)	12 (17.91)	0.498
Hypotension	5 (6.67)	2 (4.08)	0.703	5 (8.77)	2 (2.99)	0.246
Numbers of biopsy attempts, mean ± SD	2.24 ± 0.61	2.02 ± 0.48	0.027*	2.21 ± 0.59	2.10 ± 0.55	0.305

**Table 3:** Univariable and multivariable analyses for adequacy and safety outcomes

	Adequacy Outcome						Safety outcome					
	Univariable Analysis			Multivariate Analysis			Univariable Analysis			Multivariate Analysis		
	Odds ratio	95% CI	*P*-value	Odds ratio	95% CI	*P*-value	Odds ratio	95% CI	*P*-value	Odds ratio	95% CI	*P*-value
Age ≥ 65 years	0.61	0.28–1.34	0.218				0.73	0.22–2.41	0.601			
Gender, male	0.99	0.49–2.03	0.981				1.28	0.44–3.79	0.650			
Body mass index, ≥ 27 kg/m2	1.06	0.51–2.18	0.877				0.45	0.14–1.48	0.189			
Kidney length, ≥ 9 cm	1.78	0.75–4.24	0.190				0.90	0.27–3.05	0.870			
Parenchymal thickness, ≥ 15 mm	1.43	0.67–3.07	0.354				0.99	0.32–3.07	0.985			
Normal kidney echogenicity	1.98	0.88–4.44	0.097				0.16	0.02–1.25	0.080*	0.10	0.01–0.96	0.046*
Biopsy plane, longtitudinal	1.36	0.65–2.84	0.408				0.31	0.11–0.93	0.036			
Biopsy site, lower pole	0.99	0.28–3.42	0.984				0.35	0.08–1.47	0.151			
Biopsy needle direction, caudal	0.91	0.41–2.04	0.821				1.59	0.42–5.99	0.492			
Biopsy needle tangential angle, 30°–60^o^	9.11	3.73–22.24	< 0.001*	11.21	3.74–33.62	< 0.001*	0.82	0.28–2.36	0.711	0.47	0.12–1.95	0.300
Biopsy needle distance ratio												
0–0.33	5.35	0.74–38.64	0.096	6.36	0.61–66.74	0.123	1.00	reference		1.00	reference	
0.34–0.66	3.96	1.48–10.64	0.006	1.50	0.41–5.44	0.542	1.35	0.32–5.64	0.678	1.33	0.20–8.87	0.771
0.67–0.99	4.08	1.35–12.30	0.012	1.53	0.38–6.19	0.555	2.42	0.55–10.70	0.245	5.18	0.81–33.35	0.083
1	1.00	Reference		1.00	reference							
Numbers of biopsy attempts	1.42	0.75–2.68	0.285				1.36	0.59–3.11	0.469			

Regarding the safety outcome, the overall rate of major complications was 12.90%, with blood transfusion occurring in 8.06% and embolization in 3.23%. No nephrectomy or death was observed in our study. The average number of biopsy attempts was 2.15, slightly higher in the biopsy needle tangential angle of 30°–60° (2.24 vs. 2.02, *p* = 0.027). However, no statistically significant difference was observed between major complications and the biopsy needle tangential angle or the biopsy needle depth, nor with regard to biopsy needle direction or biopsy site. There was a higher rate of major complications when the biopsies were performed in the oblique plane compared to the longitudinal plane (21.28% vs. 7.79%, *p* = 0.03). However, all technical aspects became non-statistically significant after confounder adjustment, with only a traditional ultrasonographic factor of increased echogenicity retaining significance (Table 3).

## Discussion

Our study examined the technical aspects of the kidney biopsy procedure, primarily focusing on the biopsy needle tangential angle and depth for adequacy and safety outcomes. Needle trajectories with wider tangential angles (> 60°) and deeper penetration into the renal parenchyma are generally avoided due to the increased risk of the biopsy needle projecting into the renal sinus, where major vessels are located and fewer glomeruli are present. On the other hand, more superficial trajectories might yield a higher number of glomeruli, but trajectories that are too superficial or have overly narrow tangential angles (< 30°) may cause trauma to the kidney capsule, thereby increasing the risk of bleeding. Thus, an angle within 30°–60° and the middle zone of the renal parenchyma might provide a balance in yield for glomeruli with fewer complications.

Our study revealed a median number of glomeruli of 9 and tissue adequacy of 73%, which was comparatively lower than findings reported in previous studies [3, 11]. Concurrently, the observed major complication rate of 12.90% was similar to that reported in other studies [12]. This discrepancy may be attributed to the challenging nature of our enrolled patient population. Approximately 40% of the patients met the criteria for obesity based on BMI standards for Asians [10], 23% exhibited small kidney size (< 9 cm) [13], and 68% had a thin cortex (< 1.5 cm) [14], all of which may have contributed to the overall adequacy and complication outcomes. Consequently, the application of our findings may be most relevant to this high-risk population.

Our study found that biopsies performed at the tangential angle of 30°–60° had significantly better adequacy compared to angles outside that range, aligning with recent studies suggesting that a cortical tangential angle of approximately 60^o^ demonstrates higher tissue adequacy and fewer bleeding complications [3, 7]. Despite these promising findings, our study did not identify a statistically significant difference in major complication rates between the two angle ranges, except for a noted higher rate of embolization in the group with biopsy angles outside the 30°–60° range. It is essential to acknowledge that our study was not primarily designed to detect differences in complications, as it served as a secondary outcome. A comprehensive exploration of complication differences between the two angle ranges would require a larger sample size, given the rare prevalence of such events, necessitating further investigation to confirm our findings.

Regarding the depth of the needle trajectory, our study revealed a close association between the depth of the needle trajectory and the tangential angle. Wider tangential angles (> 60°) were associated with deeper needle penetration into the renal sinus (OR 8.42, *p* < 0.001) (Table 4). Notably, when compared to the group with needle trajectories passing into the renal sinus—exhibiting the lowest yield for glomeruli—it appeared that more superficial needle trajectories were associated with a higher yield for glomeruli. However, when adjusting for potential confounders, including the depth of the needle trajectory, only the biopsy angle remained significantly associated with tissue adequacy, as anticipated. Regarding safety, deeper penetration of biopsy needle trajectories was associated with higher complications, consistent with previous reports using a needle distance ratio of 0.78, representing the inner zone of the parenchyma based on our definition [7]. However, our study found that major complication rates between the biopsy needle trajectories traversing the middle and inner zones were not statistically different. Again, this might be attributed to the small sample size.

**Table 4:** Association between needle trajectory angle and trajectory biopsy zone

	Parenchymal trajectory (*N*, %)	Sinus trajectory (*N*, %)	Odds ratio (OR)	*P*-value
Angle ≤ 60°	75 (87.21%)	11 (12.79%)	Reference	NA
Angle > 60°	17 (44.74%)	21 (55.26%)	8.42	< 0.001

We acknowledge certain limitations of our study. Firstly, the retrospective nature of data collection introduces potential challenges in verifying outcomes, leading to concerns about missing data and bias. Secondly, the enrollment of a uniquely high-risk population may restrict the generalizability of our findings to broader demographic groups. Additionally, all our patients were of Asian ethnicity, known for generally smaller anatomical dimensions compared to Caucasian or African ethnicities, affecting measurements of kidney anatomy such as length or parenchymal thickness. Lastly, our study may lack sufficient power to detect differences between the proposed biopsy angles and safety outcomes. Further studies with a larger sample size would be required to confirm our results.

## Conclusion

In conclusion, our study highlights the importance of both biopsy angle and needle trajectory depth in optimizing kidney biopsy outcomes. While the biopsy angle may exert a greater influence than depth alone, the two variables are closely interrelated. Targeting the mid-parenchymal zone with an angle between 30° and 60° provides the optimal balance between diagnostic yield and safety. Although we observed no statistically significant differences in major complications, the study was not powered to detect subtle variations in complication rates.

## Data Availability

Raw study data are available from the Corresponding Author upon reasonable request.
